# What do we know from the transcriptomic studies investigating the interactions between plants and plant growth-promoting bacteria?

**DOI:** 10.3389/fpls.2022.997308

**Published:** 2022-09-15

**Authors:** Arijit Mukherjee

**Affiliations:** Department of Biology, University of Central Arkansas, Conway, AR, United States

**Keywords:** plant growth-promoting bacteria, gene expression changes, genetics, plant-microbe interaction, agriculture sustainability

## Abstract

Major crops such as corn, wheat, and rice can benefit from interactions with various plant growth-promoting bacteria (PGPB). Naturally, several studies have investigated the primary mechanisms by which these PGPB promote plant growth. These mechanisms involve biological nitrogen fixation, phytohormone synthesis, protection against biotic and abiotic stresses, etc. Decades of genetic and biochemical studies in the legume-rhizobia symbiosis and arbuscular mycorrhizal symbiosis have identified a few key plant and microbial signals regulating these symbioses. Furthermore, genetic studies in legumes have identified the host genetic pathways controlling these symbioses. But, the same depth of information does not exist for the interactions between host plants and PGPB. For instance, our knowledge of the host genes and the pathways involved in these interactions is very poor. However, some transcriptomic studies have investigated the regulation of gene expression in host plants during these interactions in recent years. In this review, we discuss some of the major findings from these studies and discuss what lies ahead. Identifying the genetic pathway(s) regulating these plant-PGPB interactions will be important as we explore ways to improve crop production sustainably.

## Introduction

One of the significant challenges facing modern agriculture is to increase crop production sustainably to maintain food security for a growing global population. In addition, climate change will continue to worsen the severity of environmental stressors such as heat, salinity, and drought on global crop production (Pachauri et al., 2014). In fact, climate change already negatively affects rice production in countries like China, India, and Indonesia, which contribute more than 75% of the total rice produced globally (Ray et al., 2015; Pickson et al., 2021). Similarly, maize, wheat, and soybean production are also under severe threat from climate change (Ray et al., 2015). Furthermore, while fertilizers have contributed to improving crop yields, the negative footprints of over-fertilizer usage on the environment are well known (Ward, 2009; Yuan et al., 2018; Sainju et al., 2019). Clearly, novel approaches need to be pursued to maximize crop productivity.

In addition to nitrogen-fixing bacteria (e.g. *Sinorhizobium*, *Mesorhizobium*, *Bradyrhizobium, Frankia*), and mycorrhizal fungi, a diverse array of plant growth-promoting bacteria (PGPB) is also beneficial to plants (Glick, 2012; Santi et al., 2013). Major crops such as corn, wheat, and rice benefit from interactions with PGPB such as *Azospirillum*, *Herbaspirillum*, *Burkholderia*, *Bacillus*, etc. Extensive studies have shown that these PGPB improve plant growth *via* biological nitrogen fixation, hormone production, and protection against biotic and abiotic stressors. A good amount of information is already available on the mechanisms of plant growth promotion by PGPB (Glick, 2012; Olanrewaju et al., 2017; Backer et al., 2018). Some studies have also investigated the bacterial molecular responses during these interactions (Shidore et al., 2012; Coutinho et al., 2015; Sheibani-Tezerji et al., 2015). One area significantly lacking is our understanding of the host genetic mechanisms regulating plant-PGPB interactions. For instance, the volume of genetic studies on host plants during these interactions is negligible compared to the number of genetic studies in legume-rhizobia symbiosis and even arbuscular mycorrhizal (AM) symbiosis. Over two decades of genetic studies in model legumes such as *Medicago truncatula*, *Lotus japonicus, Glycine max*, and *Pisum sativum* identified numerous plant genes involved at different stages of the legume-rhizobia symbiosis (Roy et al., 2020). The information gained from these studies also contributed to our current knowledge of plant genes regulating AM symbiosis. As we implement ways to develop robust and sturdier crops, especially non-legume crops, the role of PGPB in modern agriculture will continue to expand. Therefore, it is imperative to invest in genetic and biochemical studies to dissect the genes and pathways involved in plant-PGPB interactions.

A few studies involving the non-legume model plant, *Arabidopsis thaliana*, and different rhizobia, have provided important insights into these interactions (Poupin et al., 2013; Poupin et al., 2016; Zhao et al., 2018; Mendez-Gomez et al., 2021; Hernandez-Reyes et al., 2022). For instance, it has been reported that the growth promotion effects of *Rhizobium* sp. IRBG74, *A. brasilense*, and *B. phytofirmans* on *A. thaliana* involve the auxin signaling pathway (Poupin et al., 2013; Zhao et al., 2018; Mendez-Gomez et al., 2021). Furthermore, a later study showed a fine regulation of hormones (auxin and ethylene) signaling and homeostasis are important for *B. phytofirmans*-induced growth in *A. thaliana* (Poupin et al., 2016). More recently, the involvement of NIN-Like Proteins (NLPs) in rhizobium-induced changes in root architecture in *A. thaliana* was reported (Hernandez-Reyes et al., 2022). While results from these studies are encouraging, similar studies investigating the interactions between PGPB and different crops need to be pursued. In recent years, there have been some studies using genetic and molecular approaches in crops such as rice, wheat, and sugarcane to investigate these interactions at the host level, which is encouraging (Chen and Zhu, 2013; Camilios-Neto et al., 2014; Chen et al., 2015; Paungfoo-Lonhienne et al., 2016; Xie et al., 2017; Rekha et al., 2018; Wu et al., 2018; Brusamarello-Santos et al., 2019; King et al., 2019; Thomas et al., 2019; Liu et al., 2020; Malviya et al., 2020; Cook et al., 2022; Guo et al., 2022; Wiggins et al., 2022; Xu et al., 2022). This mini-review focuses on key findings from some of these studies and discusses what lies ahead.

## Plant-PGPB interactions don’t seem to utilize genes from the *Common Symbiotic Pathway*

Over the last twenty years, a combination of forward and genetic approaches has identified more than one hundred and fifty genes required for legume-rhizobia symbiosis (Roy et al., 2020). Some of the earliest genes identified were those that are necessary for the initial stages of the association. These include a cation channel (*DMI1*/*POLLUX* and *CASTOR* (a Ca^2+^ channel)), a receptor-like kinase (*DMI2*/*SYMRK*), a nuclear calcium and calmodulin-dependent kinase (*DMI3*/*CCaMK*), a transcription factor (*IPD3*/*CYCLOPS*) among others. Interestingly, plant mutants in these genes were not only affected in legume-rhizobia symbiosis but also in AM symbiosis. This led to the concept of the existence of a “Common Symbiotic Pathway (CSP)” (Mukherjee and Ané, 2011; Venkateshwaran et al., 2012; Genre and Russo, 2016; Kim et al., 2019). Furthermore, some genes from the CSP are also required for actinorhizal symbiosis and ectomycorrhizal symbiosis, indicating that the CSP is common between root nodule symbiosis (legume-rhizobia symbiosis and actinorhizal symbiosis) and mycorrhization (arbuscular mycorrhization and ectomycorrhization) (Svistoonoff et al., 2014; Genre and Russo, 2016; Cope et al., 2019). Genetic studies identified mutants in orthologs of these CSP genes in rice affected in AM symbiosis, expanding the significance of this pathway (Chen et al., 2007; Chen et al., 2008; Chen et al., 2009). The availability of mutants in CSP genes in rice offers an excellent opportunity for researchers to study the role of this pathway in other interactions between non-legumes and microbes. One study reported nitrogen-fixing rhizobia, *Rhizobium leguminosarum bv. trifollii* did not utilize the CSP genes for infection and endophytic colonization of rice roots (Chen and Zhu, 2013). Another study showed that CSP genes were not required for endophytic colonization by nitrogen-fixing bacteria, *Azoarcus* sp. (Chen et al., 2015). More recently, our group showed that plant growth-promoting bacteria, *Azospirillum brasilense*, could promote rice growth independent of the CSP and penetrate the roots of the rice symbiotic mutants (Thomas et al., 2019). Results from these studies suggest that this highly conserved genetic pathway might not be involved in interactions between PGPB and their host plants. This is quite remarkable that a genetic pathway that is functionally conserved in multiple plant-microbe symbioses doesn’t play an equivalent role in plant-PGPB interactions. While additional studies with other PGPB can be conducted to validate these observations further, PGPB likely utilizes different genetic pathway(s) to interact with host plants. Therefore, specific genetic studies directed at plant-PGPB interactions need to be pursued.

## Transcriptomic studies have revealed key gene expression patterns during plant-PGPB interactions

The advances in next-generation sequencing have led to a burst of transcriptomic studies investigating host gene expression changes during plant-PGPB interactions. Most of these studies have been performed on the Nipponbare cultivar of rice (Wu et al., 2018; Brusamarello-Santos et al., 2019; King et al., 2019; Thomas et al., 2019; Cook et al., 2022; Wiggins et al., 2022). But, other rice cultivars, such as 9311, TKM9 and IR36, have also been used (Chen et al., 2015; Xie et al., 2017; Rekha et al., 2018). Besides rice, other plants such as wheat, sugarcane, and tobacco have been used in these studies (Camilios-Neto et al., 2014; Paungfoo-Lonhienne et al., 2016; Liu et al., 2020; Malviya et al., 2020; Guo et al., 2022). Compared to the hosts, there is much more variability in the PGPB used in these studies. Some of the PGPB used include *A. brasilense*, *H. seropedicae*, *B. subtilis*, *B. anthina*, *P. kururiensis*, etc. Several of these transcriptomic studies have used the PGPB-induced growth promotion as the phenotype to confirm that the plant benefits from the interaction. So, while these studies have been performed under different experimental conditions (e.g., sterile vs. non-sterile) or using different host plants or PGPB, some key gene expression trends have emerged. For instance, genes involved in defense responses, hormone signaling, and nutrient transport were differentially expressed in host plants during interactions with PGPB. Overall, these studies have laid the groundwork for future investigations to characterize the host genetic pathways regulating plant-PGPB interactions. Below is a summary of these gene expression trends identified in these studies.

### Defense and stress response genes

The expression of defense-related genes (e.g., pathogenesis-related genes, chitinases, thionins, cinnamoyl-CoA-reductases, etc.) in the host plant is one of the primary transcriptomic responses during plant-microbe interactions. While most of these genes are upregulated during plant-pathogen interactions, some studies reported repression of defense genes in host plants during interactions with beneficial microbes (Soto et al., 2009; Liang et al., 2013; Toth and Stacey, 2015; Chen et al., 2017a). Interestingly, the transcriptomic studies in plant-PGPB interactions also showed a similar expression pattern of some well-known plant defense genes. For instance, in rice, the pathogenesis-related (PR) genes are strongly induced upon inoculation with rice blast fungus, *Magnaporthe oryzae*, which causes one of the most severe diseases in rice (Kawahara et al., 2012). However, the expression of several PR genes was reduced in host plants during interactions with different PGPB (e.g., *A. brasilense*, *H. seropedicae, P. kururiensis, B. vietnamiensis*) (Brusamarello-Santos et al., 2019; King et al., 2019; Thomas et al., 2019; Wiggins et al., 2022). Similarly, other defense genes such as chitinases, thionins, cinnamoyl-CoA-reductases, and genes encoding for disease resistance were downregulated during plant-PGPB interactions (Brusamarello-Santos et al., 2019; Thomas et al., 2019; Wiggins et al., 2022). The expression pattern of these genes supports the concept that the host plant is adjusting its transcriptional responses to accommodate the beneficial microbe. However, as observed in other beneficial plant-microbe interactions, the host defense responses were not entirely suppressed. Some defense genes, including transcription factors from the WRKY family, were also upregulated during plant-PGPB interactions (Brusamarello-Santos et al., 2019; Thomas et al., 2019; Wiggins et al., 2022). This likely allows the plant to regulate the level of microbial colonization to maintain the benefits of the interaction. Nevertheless, the reduced expression of some defense genes can be considered an important molecular response by the host plant during interactions with PGPB.

### Genes from the flavonoid synthesis pathway

Flavonoids play vital roles during different plant-microbe symbioses, such as legume-rhizobia symbiosis, AM symbiosis, and actinorhizal symbiosis (Abdel-Lateif et al., 2012; Hassan and Mathesius, 2012; Ng et al., 2015). In legume-rhizobia symbiosis and AM symbiosis it is well established that flavonoids play an integral role in the initial signal exchange between the two symbiotic partners. Some studies also implicated their potential involvement during plant-PGPB interactions (Gough et al., 1997; Webster et al., 2002). Furthermore, transcriptomic studies in rice identified several genes from the flavonoid synthesis pathway, such as chalcone synthesis genes, flavonol synthase genes, and naringenin synthesis genes differentially expressed during interactions with *A. brasilense* and *H. seropedicae* (Brusamarello-Santos et al., 2019; Thomas et al., 2019; Wiggins et al., 2022). Similarly, isoflavone reductase and phenylalanine ammonia-lyase (PAL) genes were differentially expressed during interactions with *A. brasilense* and *H. seropedicae*. These genes are also expressed during the early stages of legume-rhizobia symbiosis and AM symbiosis (Blilou et al., 2000; Chen et al., 2017b). Some flavonoid genes were also differentially expressed in wheat roots colonized by *A. brasilense* (Camilios-Neto et al., 2014). Future genetic and biochemical studies can confirm the role of the flavonoid pathway genes as manipulating this critical pathway can be an option to improve host-PGPB interactions.

### Genes encoding protein kinases

Receptor-like kinases (RLKs) play key roles in plant growth, defense responses, and plant-microbe symbioses (Li and Tax, 2013; Antolin-Llovera et al., 2014). Studies in plant disease resistance and mutualistic symbioses have shown that some RLKs are involved in the perception of microbial signaling molecules. For instance, the plant cell wall-associated kinases (WAKs) have been implicated as important regulators of plant immunity against bacterial and fungal pathogens (Kanneganti and Gupta, 2008; Amsbury, 2020). Several WAKs were differentially expressed in rice during interactions with *A. brasilense* and *H. seropedicae* (Brusamarello-Santos et al., 2019; Thomas et al., 2019; Wiggins et al., 2022). Another well-characterized group of RLKs is the Lysin motif (LysM) RLKs that play important roles in the perception of microbial signals during legume-rhizobia symbiosis and AM symbiosis (Buendia et al., 2018; Gough et al., 2018). Interestingly, the rice ortholog of the *M. truncatula NFP* gene was upregulated in expression in rice during interactions with *A. brasilense* (Thomas et al., 2019). Further studies can clarify the role of this gene during plant-PGPB interactions. Another class of receptor kinases, SHR5 RLKs, was differentially expressed in rice and sugarcane during interactions with PGPB such as *A. brasilense*, *H. seropedicae*, and *Gluconacetobacter diazotrophicus* (Vinagre et al., 2005; Brusamarello-Santos et al., 2019; Thomas et al., 2019; Wiggins et al., 2022). Because of their roles in the host plant’s response to pathogenic and beneficial microbes, RLKs are likely to be involved in plant-PGPB interactions. Identifying the PGPB-secreted signals and their corresponding plant receptors will be crucial to characterizing these interactions.

### Genes involved in hormone signaling

Major plant hormones such as auxin, ethylene, and cytokinin play key regulatory roles during plant growth and plant-microbe interactions, including pathogenic and beneficial interactions (Mukherjee and Ané, 2011; Ferguson and Mathesius, 2014; Ma and Ma, 2016). One of the proposed mechanisms by which PGPB promotes plant growth is *via* the production of auxin, 1-aminocyclopropane-1carboxylate (ACC) deaminase, cytokinin, etc. (Olanrewaju et al., 2017). Several auxin-responsive genes (e.g., *SAUR* genes, *Aux/IAA* genes, *GH3* genes) were differentially expressed in rice and wheat during interactions with different PGPB (Camilios-Neto et al., 2014; Chen et al., 2015; Xie et al., 2017; Rekha et al., 2018; Brusamarello-Santos et al., 2019; Thomas et al., 2019; Shinjo et al., 2020; Wiggins et al., 2022). Interestingly, some of these genes were suppressed in expression during these interactions. One plausible explanation for this observation is that the PGPB-synthesized auxin contributes to the endogenous hormone level in the host plant leading it to adjust the hormone level accordingly to maximize plant growth. Besides auxin, ethylene plays an integral role in plant-microbe interactions. Ethylene levels are increased in response to abiotic and biotic stresses, including fungal and bacterial pathogens, leading to the inhibition of plant growth (Olanrewaju et al., 2017). The ACC oxidase enzyme is an essential catalyst in the ethylene biosynthetic pathway (Houben and Van de Poel, 2019). Gene expression studies in rice identified several ACC genes downregulated in expression during interactions with *A. brasilense* and *H. seropedicae* (Brusamarello-Santos et al., 2019; Thomas et al., 2019; Wiggins et al., 2022). A similar expression pattern of these genes was also detected in wheat during interactions with *A. brasilense* (Camilios-Neto et al., 2014). These results suggest that the host plant reduces its ethylene levels by reducing ACC levels in response to the beneficial PGPB. The expression data further supports a mechanism proposed earlier in which some PGPB secrete the ACC deaminase enzyme, which reduces ethylene production in host plants by degrading ACC (Olanrewaju et al., 2017). However, genetic studies still need to be performed with plant and PGPB mutants affected in hormonal pathways to confirm the role of these hormones in PGPB-induced growth.

### Genes involved in nutrient uptake

Studies have shown nitrate and ammonium transporters regulate plant growth and nitrogen use efficiency (Courty et al., 2015; Fan et al., 2017). Since one of the primary mechanisms by which PGPB promote plant growth is *via* improved nitrogen uptake, it is not unrealistic to expect these transporters to play important roles in plant-PGPB interactions. Transcriptomic studies revealed nitrate and ammonium transporters were differentially expressed in rice during interactions with *A. brasilense* and *H. seropedicae* (Thomas et al., 2019; Wiggins et al., 2022). Another study reported nitrate transporters were differentially expressed in wheat roots colonized by *A. brasilense* (Camilios-Neto et al., 2014). The nitrate transporters are excellent reporter genes for these interactions as their expression suggests that the PGPB is likely facilitating improved nitrate uptake in the host plant. Nitrate reductases also play important roles in nitrogen uptake by plants (Campbell, 1999). Some studies have reported the expression of nitrate reductases in root nodules of legumes (Kato et al., 2003; Horchani et al., 2011). Genes encoding nitrate reductases were differentially expressed in rice during interactions with *A. brasilense* and *H. seropedicae* (Thomas et al., 2019; Wiggins et al., 2022). During mutualistic plant-microbe interactions, the symbiotic microbes benefit from receiving carbohydrates from the host plants *via* sugar transporters (Manck-Gotzenberger and Requena, 2016; Sugiyama et al., 2017; Bezrutczyk et al., 2018). These transporters were differentially expressed in rice roots during interactions with *A. brasilense* and *H. seropedicae* (Thomas et al., 2019; Wiggins et al., 2022). Sucrose synthases play important roles in sugar metabolism and have been suggested to be involved in delivering photosynthates to microbial symbionts (Gordon et al., 1999; Silvente et al., 2003). In sugarcane, sucrose synthases were upregulated in expression during interactions with *Burkholderia* Q208 (Paungfoo-Lonhienne et al., 2016). Overall, the differential expression of the nitrate and sugar transporters are excellent indicators of effective mutualistic interaction between the host plant and the symbiotic microbe.

## Conclusions and perspectives

Major crops have been known to benefit from interactions with PGPB for a long time. Although some of these bacterial strains are commercialized, PGPB-inoculated crops still represent a small fraction of crops grown globally. To maximize the potential of these interactions in global crop production, it is essential to characterize these interactions mechanistically. While several transcriptomic studies have aided in identifying plant genes and genetic pathways likely to be involved in these interactions, it is imperative to follow up with rigorous genetic and biochemical analyses to confirm that. One of the challenges in studying these interactions at a genetic level is the lack of host-symbiont specificity. In legume-rhizobia symbiosis, where the interaction is very host-specific, it was reasonably straightforward to proceed with model plants like *Medicago truncatula*, *Lotus japonicus, Glycine max*, and *Pisum sativum*. However, most PGPB have a diverse host range that includes non-legume crops such as rice, maize, sugarcane, wheat, etc. While the wide range of host plants that benefit from PGPB is an advantage of these interactions, it creates a dilemma when selecting the model plant system for genetic studies. Regarding the host, rice seems to be an excellent candidate because of the availability of numerous genetic resources. It is no surprise that most transcriptomic studies on plant-PGPB interactions have been performed on rice. However, it will also be essential to conduct similar studies in other plants (e.g., wheat, sugarcane, maize) to accelerate the process of gene discovery or identify host-specific genes or pathways. *Setaria viridis*, green foxtail millet, has also been proposed to be used as a model plant to study these interactions genetically (Pankievicz et al., 2021). Several studies investigating these interactions have also used the model plant *A*. *thaliana* (Poupin et al., 2013; Poupin et al., 2016; Zhao et al., 2018; Mendez-Gomez et al., 2021; Hernandez-Reyes et al., 2022). In the transcriptomic studies performed so far, a wide range of PGPB strains have been used. Proceeding with one PGPB strain can make the studies more focused and accelerate the identification of genetic pathways relevant to that interaction. However, with numerous agriculturally relevant PGPB strains available, selecting only one PGPB strain for genetic studies will be shortsighted. Studies in other plant-microbe symbioses identified and purified the microbial signals, which are also commercialized for agricultural purposes. Future studies can identify PGPB-secreted signaling molecules with plant growth promotion effects. One study has already reported that diffusible and volatile compounds secreted by *A. brasilense* could promote growth in *A. thaliana*, which is promising (Mendez-Gomez et al., 2021). Most plant-PGPB studies have focused on rhizospheric bacteria. However, to gain better clarity of these systems, it will also be essential to address the microbial metagenome in seeds and roots. As is evident, there are plenty of opportunities to conduct genetic and biochemical studies that will fill the gaps in our understanding of these important plant-microbe interactions. With climate change continuing to pose significant challenges for crop productivity in the future, exploiting the plant-PGPB interactions seems like a perfect opportunity to improve crop growth sustainably.

## Author contributions

AM wrote the mini-review. The author confirms being the sole contributor of this work and has approved it for publication.

## Funding

This work was financially supported by the Arkansas INBRE program, supported by a grant from the National Institute of General Medical Sciences, (NIGMS), P20 GM103429 from the National Institutes of Health.

## Acknowledgments

I thank the reviewers for their helpful suggestions and critique of the manuscript. I also apologize to colleagues whose work was not cited due to space limitations or the focus of this mini-review.

## Conflict of interest

The author declares that the research was conducted in the absence of any commercial or financial relationships that could be construed as a potential conflict of interest.

## Publisher’s note

All claims expressed in this article are solely those of the authors and do not necessarily represent those of their affiliated organizations, or those of the publisher, the editors and the reviewers. Any product that may be evaluated in this article, or claim that may be made by its manufacturer, is not guaranteed or endorsed by the publisher.
